# Insight into the Evolution of the Histidine Triad Protein (HTP) Family in *Streptococcus*

**DOI:** 10.1371/journal.pone.0060116

**Published:** 2013-03-20

**Authors:** Zhu-Qing Shao, Yan-Mei Zhang, Xiu-Zhen Pan, Bin Wang, Jian-Qun Chen

**Affiliations:** 1 State Key Laboratory of Pharmaceutical Biotechnology, School of Life Sciences, Nanjing University, Nanjing, Jiangsu Province, China; 2 Department of Epidemiology, Research Institute for Medicine of Nanjing Command, Nanjing, China; University Medical Center Utrecht, The Netherlands

## Abstract

The Histidine Triad Proteins (HTPs), also known as Pht proteins in *Streptococcus pneumoniae*, constitute a family of surface-exposed proteins that exist in many pathogenic streptococcal species. Although many studies have revealed the importance of HTPs in streptococcal physiology and pathogenicity, little is known about their origin and evolution. In this study, after identifying all *htp* homologs from 105 streptococcal genomes representing 38 different species/subspecies, we analyzed their domain structures, positions in genome, and most importantly, their evolutionary histories. By further projecting this information onto the streptococcal phylogeny, we made several major findings. First, *htp* genes originated earlier than the *Streptococcus* genus and gene-loss events have occurred among three streptococcal groups, resulting in the absence of the *htp* gene in the Bovis, Mutans and Salivarius groups. Second, the copy number of *htp* genes in other groups of *Streptococcus* is variable, ranging from one to four functional copies. Third, both phylogenetic evidence and domain structure analyses support the division of two *htp* subfamilies, designated as *htp* I and *htp* II. Although present mainly in the pyogenic group and in *Streptococcus suis*, *htp* II members are distinct from *htp* I due to the presence of an additional leucine-rich-repeat domain at the C-terminus. Finally, *htp* genes exhibit a faster nucleotide substitution rate than do housekeeping genes. Specifically, the regions outside the HTP domains are under strong positive selection. This distinct evolutionary pattern likely helped *Streptococcus* to easily escape from recognition by host immunity.

## Introduction

The genus *Streptococcus* comprises a wide variety of pathogenic and commensal gram-positive bacteria [1]. Among them, *Streptococcus pyogenes*, *Streptococcus agalactiae* and *Streptococcus pneumoniae* are the three most important streptococcal human pathogens [2]–[5]. Despite sharing the same hosts and showing similar pathogenicity, the genomes of these three species showed only a modicum of similarity. With dramatic gene gains and losses, chromosomal rearrangements and horizontal gene transfers having frequently occurred since their separation, the core-genome of these three streptococci only contains about 50% of the genes of each species [4]–[12]. As a result, it is difficult to identify virulence factors that are shared by all three streptococci; and many currently defined virulence-related proteins are species-specific [13]–[15].

However, several recent studies revealed that a family of proteins characterized by repeated histidine triad motifs (His-x-x-His-x-His) is present in all three human-pathogenic streptococci, and these proteins play roles in pathogenesis [16]–[18]. Proteins belonging to this family were first discovered in *S. pneumoniae* and were given different names by independent research groups, such as Pht (for pneumococcal histidine triad) [19], Php (for pneumococcal histidine protein) [20] and BVH [21]. Adamou *et al.* [19] found that although the sequence identity among four *pht* genes in *S. pneumoniae* varied from only 32% to 87%, a typical character of 4–6 duplicated histidine triad motifs was consistently shared by each protein. Searching for this signature, researchers subsequently identified genes homologous to *pht* genes in two other human pathogens, *S. pyogenes* [18], [22] and *S. agalactiae* [23]; and also in the zoonotic pathogen *S. suis* [24], [25]. The identified *pht* homologs were named, *htp*A and *slr* in *S. pyogenes*; *sht and blr* in *S. agalactiae*; and *htpS* in *S. suis*. For convenience, we hereafter refer to these genes as histidine triad protein (*htp*) genes, and the conserved histidine triad motifs as the HTP domain.

HTPs are a family of cell-surface exposed proteins that are involved in a diverse range of important biological functions [26]. The crystal structure of a PhtA protein fragment has revealed that the HTP domain forms a zinc-binding fold [27], [28]. The Zn^2+^ binding ability of HTP proteins was indeed confirmed for HtpA and PhtD by independent groups [18], [29]. Bioinformatic analysis further revealed that a cis-element recognized by AdcR, a zinc uptake regulator, often exists in the promoter region of these *htp* genes [30]. Recent experiments in both *S. pneumoniae* and *S. suis* confirmed that *htp* genes were regulated by AdcR [16], [25]. Furthermore, parallel studies in *S. pneumoniae* and *S. agalactiae* both support the concept that HTP proteins are involved in bacterial evasion of complement-mediated host immune responses by binding to complement factor H [16], [17]. The fusion of the HTP domain with a leucine-rich-repeat (LRR) domain was also identified [22], [23]. This new type of chimeric protein in *S. pyogenes* was recently reported as an adhesion protein binding to human Type I Collagen [31]. In addition, investigators have tried to introduce HTP as a subunit of multivalent vaccines against streptococcal infection. As a family of surface-exposed proteins that are expressed during the bacterial infection process, HTP homologs have been proven to possess strong immunogenicity and shown to induce specific humoral immunity in the host [24], [32]–[34]. Several studies have suggested that immunization with recombinant proteins of HTP homologs from different streptococcal species can prevent bacterial infection in mice [18]–[21], [24]. More importantly, immunization with PhtD also successfully confers protection against *S. pneumoniae* in rhesus monkeys, indicating its potential application to humans [35].

In contrast to extensive studies on the biological functions and potential clinical applications of HTP proteins, little attention has been paid to the origin and evolution of this family. Interestingly, all currently investigated *htp* genes are encoded by pathogenic streptococcal species. Whether this family of proteins also exists in non-pathogenic *Streptococcus* or other bacteria is still unknown. With the development of sequencing technology, many streptococcal genomes have been fully sequenced [36]. Herein, we investigated the distribution of *htp* genes among 105 streptococcal genomes representing 38 different species/subspecies, explored the origin and evolutionary history of the *htp* gene family among different streptococcal groups, and discovered the distinct evolutionary patterns of this cell surface-exposed protein family.

## Results

### Reconstructing the Phylogenetic Tree of *Streptococcus*

**Figure 1:**
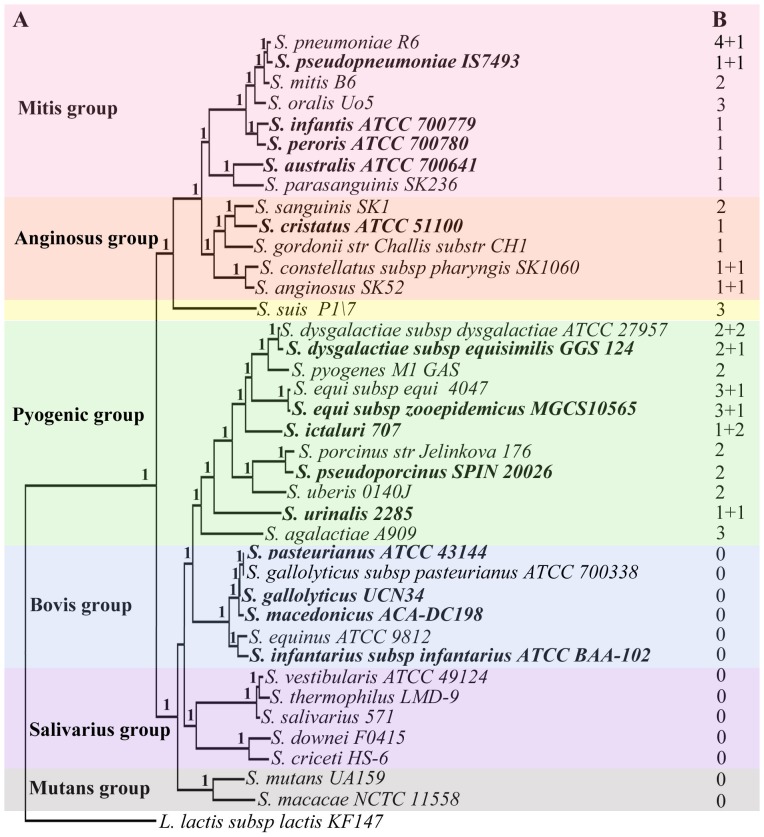
Distribution of all identified *htp* genes among 38 streptococci in a clear phylogenetic background. A. Phylogenetic analysis of 38 streptococci based on 530 single-copy orthologous proteins. Node labels indicate aLRT SH-like branch support values. Twenty-six species have been used in the previous phylogeny analysis based on 16s rDNA, while 14 species or subspecies were newly added and labeled in bold. Group division was updated based on the phylogenetic relationship. B. Copy numbers of *htp* genes within each genome are indicated with complete genes plus pseudogenes.

To elucidate the evolutionary history of *htp* genes, it is necessary to determine the phylogenetic relationships within the genus *Streptococcus*. Based solely on 16S rDNA sequences, a previous study [1] divided streptococcal species into six groups (Anginosus, Bovis, Mitis, Mutans, Pyogenic, and Salivarius). Herein, based on 530 single-copy orthologous gene sequences extracted from representative genomes of 38 streptococcal species/subspecies and also an out-group species *Lactococcus lactis*, we were able to generate a 192,452 long alignment matrix and to reconstruct a well-supported species tree for *Streptococcus*. The entire tree encompasses 38 taxa in total, with fourteen newly added. As shown in Figure 1A, by using *Lactococcus lactis* as an out-group species, two major clades were identified with strong supports. One clade comprises the Anginosus group, Mitis group and *S. suis* (the latter being a species that has heretofore not been assigned to any group). Another streptococcal clade consists of four other groups (Salivarius, Mutans, Bovis, and Pyogenic). Overall, the phylogenetic tree we obtained (Figure 1A) was consistent with the previous 16S tree [1], [4]. However, there were also two differences. In the previous 16S tree [1], the *S. sanguinis* and *S. gordonii* species were placed in the monophyletic Mitis group, whereas in our tree these two species plus the newly added species *S. cristatus* were found, with high support, in Anginosus group (sister lineage of the Mitis group). The second inconsistency between our tree and the 16S tree is the separation of four previously defined Mutans group species into two independent lineages. As shown in Figure 1A, the two species *S. criceti* and *S. downei* that were previously placed in the monophyletic Mutans group were now separated from this lineage and clustered in the Salivarius group.

### Identification of *htp* Genes in *Streptococcus*

By performing BLAST and HMM searches, we were able to identify a total of 240 putative *htp* genes from 84 out of 105 surveyed streptococcal genomes, covering 25 out of 38 species/subspecies. No *htp* gene was found from the rest 21 surveyed genomes representing thirteen streptococcal species.

The length of those identified sequences was highly variable. The shortest sequence found in *S. pneumoniae* comprised only 138 nucleotides, with only one HTP domain found in its deduced amino acid sequence. In contrast, the longest gene found in *S. mitis* codes for a protein of 1327 amino acids in length and has nine HTP domains. Also, many short genes appeared in tandem with very short intervals. As described in several previously published literatures [19], [24], *htp* genes usually encode proteins with more than 800 amino acids. Therefore, we speculated that these short genes are likely mis-annotated. According to a previous study [37], the rate of automated annotation error is about 36% in a plant genome. One important obstacle to automated annotation is reading frame shift, caused by either spontaneous mutation or sequence error. Automated annotation would not identify the sequence as a pseudogene on the occasion of reading frame shift, but divide the sequence into two coding exons by insetting an intron in eukaryotic genomes or factitiously generating two genes that actually do not exist. Considering this, we undertook manual re-annotation and analysis for all identified genes shorter than 2 kb. For the genes that appeared in pair, both of the two fragments were subjected to Blastn search against a local database comprised of genes longer than 2 kb. If the two genes both had high similarity to one long gene but localized in different regions, they were regarded as one pseudogene. For other short genes, ∼500 bp of both the 5′ and 3′ flanking sequences were analyzed to evaluate whether they are long *htp* genes. If reading frame shifts or non-sense mutations were observed, then the gene was also identified as a pseudogene. A total of 190 intact *htp* genes were finally obtained.

The number of intact *htp* genes found from each surveyed genome is listed in Table S1. From this table, one could tell that the copy number of *htp* genes was largely consistent among different isolates of a streptococcal species. For example, two conserved *htp* genes were consistently found in all 14 *S. pyogenes* isolates surveyed and three copies were always detected in 13 *S. suis* isolates. However, differently in *S. pneumoniae* and *S. agalactiae*, the intact *htp* gene copy number was variable among different isolates, ranging from two to four and two to three copies, respectively.

To avoid redundant calculations and to maximally represent the diversity of streptococcal *htp* genes for further analyses, we selected one genome with maximal copy of *htp* gene to represent each species/subspecies (Table S1). For *S. pneumoniae* and *S. agalactiae*, we selected the isolates *S. pneumoniae* R6 and *S. agalactiae* A909, since they contain the maximal number of *htp* genes in the two species. As a result, from a total of 38 representative genomes, we finally identified 46 intact *htp* genes as well as 12 pseudo ones (Figure 1), exhibiting a concise picture for *htp* gene distributions in *Streptococcus*.

### Variant *htp* Genes in Streptococcal Species

As shown in Figure 1, the 46 functional *htp* genes were identified from 25 representative streptococcal genomes (representing Mitis, Anginosus, Pyogenic groups and also *S. suis*), but the *htp* gene was not found in the other thirteen species (representing Bovis, Mutans and Salivarius groups); this indicated an unequal distribution of *htp* genes among the different groups. We also found that among the 25 species with *htp* genes, the maximal copy number of *htp* genes was variable, ranging from one to four copies. After locating the *htp* genes on the chromosome of each species (Figure S1), we found that a majority of *htp* genes was maintained in one of the two conserved gene contexts: in the first case, many *htp* genes formed an operon with an upstream laminin-binding protein gene (*lmb*, also termed *lbp*) [18], [19], [24]. Among the 46 *htp* genes, 26 appeared in the *lmb-htp* operon structure (Figure S1). In the second case, some *htp* genes were located between two conserved housekeeping genes, and interestingly, these *htp* genes all had a LRR domain at the C-terminus, forming an *htp-lrr* gene structure (Figure S1). Also, this *htp-lrr* type gene appears to mainly exist in Pyogenic group species, as it was missing from species of the Mitis and Anginosus groups. In addition, there were also some remaining *htp* genes located in non-conserved regions; and some were accompanied by transposons (Figure S1).

### Identification of *htp* Genes from Bacteria Outside *Streptococcus*

Previously, it was speculated that *htp* genes only exist in the genus *Streptococcus* [38]. To test this hypothesis, we performed an online Blastp search against over 1800 non-streptococcal bacterial genomes in the Genbank database with the E-value set to 10. After manually checking and subjecting the hits to Pfam analysis, a total of 10 *htp* genes were surprisingly found to be in nine other bacteria. The genome of the *Granulicatella elegans* strain ATCC 700633 contained two *htp* genes, whereas the other eight genomes including *Gemella sanguinis* M325, *Gemella haemolysans* M341, *Gemella haemolysans* ATCC 10379, *Slackia exigua* ATCC 700122, *Granulicatella adiacens* ATCC 49175, *Catonella morbi* ATCC 51271, *Aerococcus urinae* ACS120VCol10a and *Facklamia languida* CCUG 37842 each had one *htp* gene. Among these species, seven of them (like *Streptococcus*), belong to the class Bacilli within the phylum Firmicutes; the remaining two species *Catonella morbi* and *Slackia exigua* belong to class Clostridia within the phyla Firmicutes and Actinobacteria, respectively. Among these *htp* genes identified from non-streptococcal species, two appeared in *lmb-htp* formats; and the one in *Slackia exigua* was located downstream of *adc*A, which is a homolog of *lmb*. The rest of the seven *htp* genes were not found in a conserved gene context compared with *Streptococcus*.

### Inferring the Evolutionary History of *htp* Genes

A typical character of the *htp* gene is that it encodes one to multiple HTP domains. However in some *htp* genes, the LRR domains were found at the C-terminus; which has been previously reported in *S. agalactiae* and *S. pyogenes* [22], [23]. Our data revealed that among the 46 functional *htp* genes in *Streptococcus*, ten showed this chimeric structure (Figure S1). Furthermore, the *htp*-*lrr* type gene was also found in *G. haemolysans*, a non-streptococcal species.

**Figure 2:**
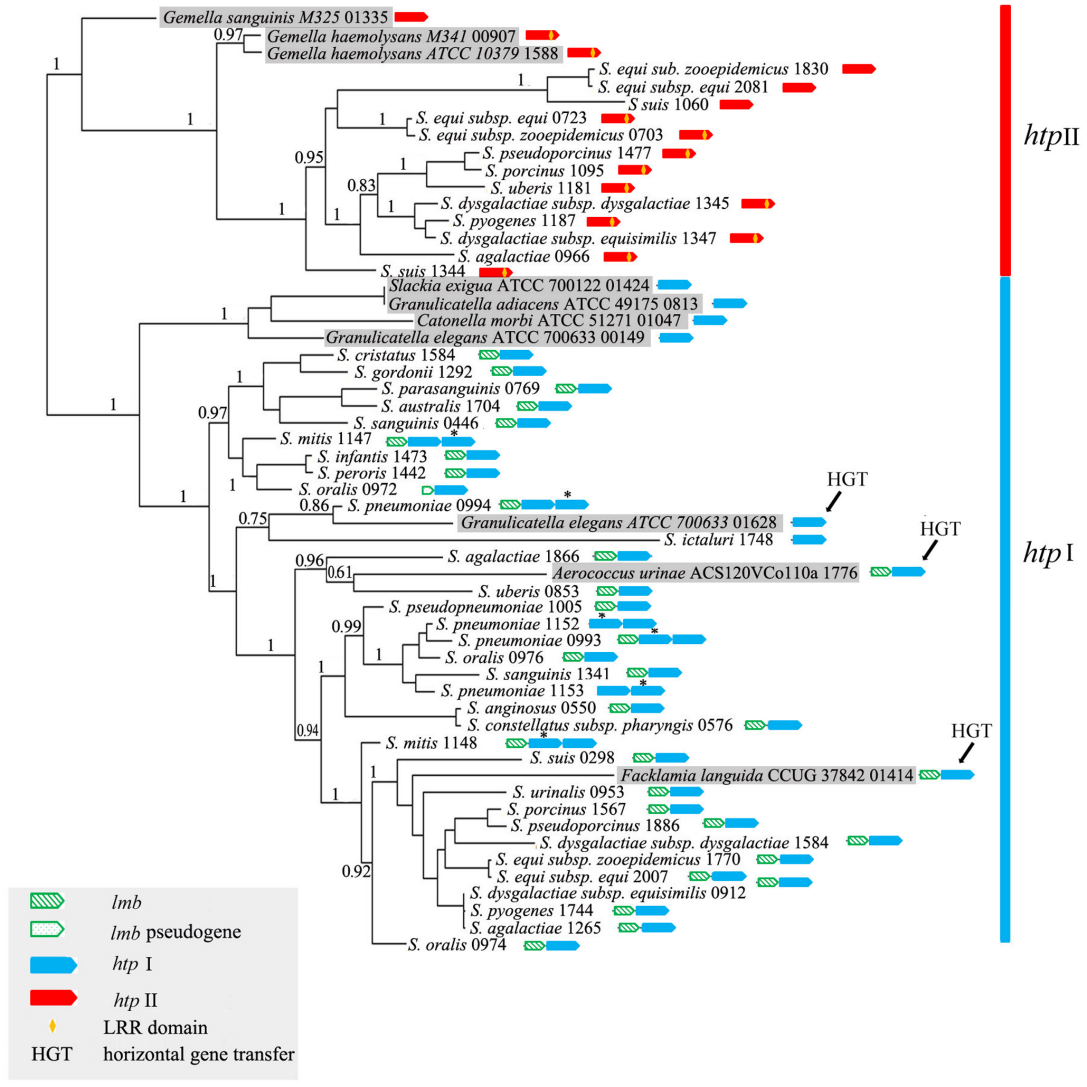
The phylogenetic tree of *htp* genes based on CDS. Node labels indicate aLRT SH-like branch support values. The *htp* genes found from non-streptococcal species are shaded. For each gene, the CDS number in its genome is found behind the species name. Arrows in different colors are used to represent *htp* I or *htp* II genes. Asterisks (*) are used to indicate the represented gene when a species has tandem duplicated *htp* genes.

In order to determine whether *htp* genes obtained in non-streptococcal species were recently acquired from streptococcal species by horizontal transfer, and to understand the origin and evolutionary history of *htp* genes, two phylogenetic trees were reconstructed based on either CDS or amino acid sequences of 56 *htp* genes identified from the representative streptococcal genomes and other bacteria. These two trees showed very similar topologies and are supportive of the theory that *htp* genes formed into two clades or subfamilies (Figures 2 and S2). Sixteen genes formed a small subfamily, with all *htp*-*lrr* type genes included; whereas the other 40 *htp* sequences formed a large subfamily and none possessed a LRR domain. We designated the large subfamily as subfamily I (*htp* I), and the small subfamily that contains *htp*-*lrr* type genes as subfamily II (*htp* II).

Importantly, both subfamilies included sequences from non-streptococcal species. In subfamily II, three sequences from *G. sanguinis* and *G. haemolysans* formed the basal lineages of this clade, while four sequences from *S. exigua*, *G. elegans*, *G. adiacens* and *C. morbi* also forming the basal lineages in subfamily I (Figure 2). This topology strongly indicated that the division of the two subfamilies must have occurred earlier than did the genus *Streptococcus*. Also within subfamily I, three *htp* genes in *F. languida*, *A. urinae* and *G. elegans* were closely related with streptococcal *htp* genes, suggesting three horizontal gene transfer events.

Ten *Streptococcus* species were found to have both *htp* I and II copies, including nine species of the Pyogenic group and also *S. suis* (Figures 2 and S1). In the *htp* II clade, two subspecies of *S.equi* and species *S. suis* were found to each possess two copies of *htp* II genes, with one copy manifesting LRR domain while the other did not. Seven other streptococcal species have only one copy of the *htp* II gene, which contains the LRR domain (Figure 2). Moreover, one non-streptococcal species, *G. haemolysans* (two strains), was found to have an *htp*-*lrr* type gene. This suggests to us that LRR domain was lost from the second copy of the *htp* II gene in subspecies of *S.equi* and the species *S. suis*.

Previously, we found that the functional *htp-lrr* type gene was lacking in both the Mitis and Anginosus groups. However, by checking pseudogenes we actually found one each in *S. anginosus* and *S. constellatus subspecies pharyngis* showing high sequence identity to *htp* II genes (Figure S1). Additionally, one functional copy of the *htp*-lrr gene was found in the species *S. suis* (which evolutionarily separated early); and in Figure 2, this *S. suis htp* II gene was indeed located outside of the Pyogenic group *htp* II genes, suggesting a consistency between gene tree and species tree. Considering these findings, we argue that one functional *htp* II gene (with LRR) likely exists in the common ancestor of all streptococcal species; however, it was pseudogenized in Anginosus group and totally lost from the Mitis group.

### Frequent Recombination and Fast Rate of Evolution of *htp* Genes

Although bacteria are haploid, recombination could happen among multi-copy genes. On the other hand, horizontal transfer of DNA fragment also provides an opportunity for recombination events to occur. To study how frequently recombination occurred among *htp* genes in *Streptococcus*, we performed recombination analysis with RDP software for 46 streptococcal *htp* genes. As shown in Figure S3, a total of 66 recombination events were detected, with many more events observed in *htp* I (58/66) than in the *htp* II subfamily (8/66). Among all the 66 recombination events, the parent sequences of 46 events were clearly detected, whereas the parent sequences of 20 other recombination events could not be distinguished unambiguously.

To evaluate the rate of evolution for *htp* genes, the synonymous substitution rates of *htp* genes within each subfamily were compared with those of neighboring *lmb* genes and eight housekeeping genes. The average Ks values for *htp* I (1.92±0.05) and *htp* II (2.06±0.1) were similar to that of *lmb* (2.13±0.06), but all were higher than the Ks of housekeeping genes (1.58±0.03); indicating that *htp* genes and the *lmb*-*htp* operon evolved faster than did housekeeping genes. Interestingly, the Ks of HTP and LRR domains (1.52±0.09, 1.24±0.11) were consistently lower than those of the overall Ks value of the entire gene, and lower even than the housekeeping gene, indicating strong mutation constraints on the evolution of these domains.

### Conservation of Functional Domains in *htp* Genes

**Figure 3:**
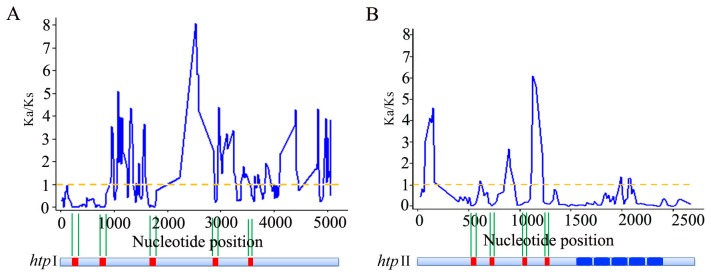
Sliding-window analysis for Ka/Ks values of two subfamilies of *htp* genes. Ka/Ks ratios were calculated for A) *htp* I subfamily and B) *htp* II subfamily using DnaSP with a sliding window of 60 bases and a 15-base step size.

In order to provide higher resolution of the nucleotide substitution patterns among different regions of *htp* genes, and especially to elucidate whether certain sites are under positive selection, a sliding Ka/Ks window analysis was carried out for two *htp* subfamilies using Dnasp v5.0 [39]. In the *htp* I subfamily, a high Ka/Ks ratio (>1) was detected throughout the coding regions (Figure 3A). However, the scattered *htp* domains showed much lower Ka/Ks ratios (<1, Figure 3A), indicating a purifying selection on these domains. Similarly, for *htp* II subfamily, all HTP domains and most LRR domains showed low Ka/Ks ratios (<1); however, regions outside of these domains often showed large Ka/Ks (>1, Figure 3B). We also detected regions in the LRR domain showing Ka/Ks ratios larger than 1 (Figure 3B). Currently it is unknown whether positive selection occurring in this LRR region would make HTP II proteins change their ligand-binding abilities so as to adapt to different hosts or tissues. This deserves further experimental study.

## Discussion

### The Early Origin and Separation of the Two *htp* gene Subfamilies

The first HTP protein was found in *S. pneumoniae* in 2001 [19], and during the next decade, additional HTP proteins were found in several pathogenic streptococci [18], [24]. However, the origin and evolution of this novel family have not been evaluated. In this study, a maximum of 46 complete *htp* genes was identified among 25 streptococcal species/subspecies. These *htp* genes could be divided into *htp* I and *htp* II subfamilies based upon their locations in a phylogenetic tree (Figure 2). Over the past ten years, it has been hypothesized that this family protein may be restricted only in the genus *Streptococcus* [38]. In this study, we are able to identify 10 *htp* genes from nine non-streptococcal bacteria. Because all of these bacteria can infect humans, we determined whether the *htp* genes found in their genomes were a result of recent horizontal gene transfer events. Our phylogenetic analysis revealed that although three of the *htp* genes were indeed acquired from some streptococci by horizontal gene transfer, others fell outside of the streptococcal *htp* genes for both the *htp* I and *htp* II clades, precluding the possibility of recent lateral acquisition of *htp* genes in these seven non-streptococcal species via horizontal gene transfer. These findings offer strong evidence to support the notion that *htp* genes originated earlier than did the *Streptococcus* genus. The separation of *htp* I and *htp* II genes for both streptococcal and non-streptococcal species also indicates that both *htp* I and II genes appeared in the common ancestor of *Streptococcus*.

As for *htp* gene location, an interesting finding was that many *htp* I genes were present in *lmb*-*htp* operon and many *htp* II genes were located between two conserved housekeeping genes. Such conservation in gene context further suggested to us the ancientness of the two *htp* subfamilies. We conjecture that in the common ancestor of *Streptococcus*, one copy of the *htp* I gene was involved in the *lmb*-*htp* operon with its own promoter region lost evolutionarily; while the other copy of the *htp* II gene fused with a LRR domain, as the out-group species *G. haemolysans* demonstrated.

### The Evolutionary History of *htp* Genes in *Streptococcus*

Although the origin and separation of *htp* genes appeared to have occurred earlier than did *Streptococcus*, both *htp* subfamily genes were evidently lost later in three groups of *Streptococcus* (Bovis, Mutans and Salivarius). Since both *htp* I and II subfamilies are present in later-derived Pyogenic group and also in the individual species *S. suis*, the absence of *htp* genes in these groups is likely due to independent gene-loss events that occurred during lineage-specific evolution. Likewise, the *htp* II gene appeared to be totally absent among eight Mitis group species, whereas two *htp* II pseudogenes were still found in two of five closely related Anginosus group species (Figure S1). This strongly supports the concept that the *htp* II subfamily was lost from the Mitis group after separating from the Anginosus group.

Gene duplication and deletion were also frequently observed in the *Streptococcus* pyogenic group. However, both subfamily genes are well maintained in their genomes, which indicates that both types of genes are functionally important to these bacteria. Indeed, previous studies have shown that both subfamily genes play important roles in the survival and/or pathogenic processes of these bacteria [18], [22], [23], [40]. Knocking out either the *htp* I subfamily gene *htp*A or the *htp* II subfamily gene *slr* significantly decreased the pathogenicity of *S. pyogenes* [22]. However, the mechanism(s) underlining this phenomenon is still unclear. Some investigators have proposed that the decreased pathogenicity of mutated *S. pyogenes* results from a reduced capability of zinc absorption [18]. Other recent studies carried out by Bober *et al.* [31] demonstrated that Slr could adhere to collagen I with high affinity, which is critical for colonization and infection of *S. pyogenes*. Functional differentiation between *htp* I and *htp* II subfamilies may result from the absence or presence of LRR domains in their C-terminus.

Phylogenetic and chromosomal synteny analysis revealed that gene birth and death processes frequently occur across species. Tandem or segmental duplication has contributed to the increase in copy number in many species. As shown in Figure S2, although it does not possess an *htp* II gene, *S. pneumoniae* contains four functional *htp* I genes and one *htp* I pseudogene (incorrectly annotated as five tandem ORFs). By comparing *S. pneumoniae* with its closely related species within the same group, we observed that *htp* genes have experienced several rounds of gene duplications. These duplicated genes likely play redundant roles in *S. pneumoniae*, because variation of *htp* gene copy number was found among *S. pneumoniae* isolates (Table S1). Furthermore, it was also found that independently knocking out three of the four functional *htp* genes did not affect its pathogenicity [16]. The dynamic birth and death evolution of *htp* genes of this lineage could potentially provide opportunities for newer functional innovations.

### Sophisticated Evolutionary Strategies Drive HTP Proteins to Escape from Host Immune System Recognition

The synonymous substitution rates of both *htp* I and *htp* II genes were greater than those for housekeeping genes, suggesting a fast rate of evolution for *htp* gene. However, the Ks value of the HTP and LRR domains were even lower than those of housekeeping gene, reflecting strict mutation constraints on these domains. Increase evidence supports the hypothesis that synonymous substitutions suffer selections from codon usage or order bias [41], [42]; however, whether this inconsistent substitution rate within the same gene is caused by selection or mutation bias is unknown. HTP proteins are involved in several important functions of *Streptococcus*, including zinc absorption, binding of complement H and adhesion to extracellular matrix. Although gene structures between the two *htp* subfamilies are different, they both share the HTP domain. Crystal structure analysis revealed that the property of zinc binding is subserved by the HTP domain [28]. Recent study by Loisel *et al.* [29] found that zinc-binding capability is positively correlated with the total number of HTP domains. In addition, a conserved AdcR binding element was found in promoter regions of almost all identified *htp* genes. As shown in Figure 1, many pathogenic species such as *S. pneumoniae*, *S. agalactiae* and *S. pyogenes* possessed at least one *htp* gene, whereas a complete loss of the *htp* gene was only observed in some commensal and non-pathogenic streptococcal species, such as *S. salivarius* and *S. thermophilus*. This correlation indicates a role of *htp* genes in *Streptococcus* infection.

Proteins secreted to the cell wall of bacteria are easily detected by the immune system, and are therefore likely to encounter positive selection. A recent study in *S. pneumoniae* found that genes encoding surface antigens are often under diversifying selection so as to escape targeting by the human immune system [43]. This phenomenon has also been shown for other animal and plant pathogens [44]–[49]. A study on the surface exposed toxin protein VacA of *Helicobacter pylori* found that all sites under positive selection of this protein were located on the surface of its tertiary structure, and speculated that these residues are subjected to antibody recognition [50]. Our data revealed that the *htp* gene is also under positive selection. Sliding window of Ka/Ks analysis demonstrated that in *htp* genes, regions outside the HTP or LRR domains are often subject to strong positive selection (Figure 3); while HTP or LRR domains often exhibit low Ka/Ks values. These opposite evolutionary patterns observed within and outside functional domains of *htp* genes likely reflect a sophisticated strategy in the evolution of these bacterial cell surface-exposed proteins. These findings from our study and from others suggested that positive selection with respect to surface proteins is not a rare occurrence, but is rather ubiquitous in bacterial pathogens. A comprehensive study published recently by Nogueira *et al.* [51] reported that in bacteria cell-exposed and secreted proteins generally have a fast rate of evolution and are often under positive selection. Additionally, the frequent recombination events observed in this study may also be due to a selective advantage that further increases the sequence divergences. Collectively, two opposite forces are coordinated in shaping the evolution of *htp* genes: negative selection pressure that maintain the conserved functions (such as Zinc absorption, adhesion, *etc*.) of HTP and/or LRR domains; and positive selection pressure which comes from host immune system and drive the quick evolution of non-essential regions to change their amino acids; this would then assist the bacteria in escaping recognition by the host immune system.

## Materials and Methods

### Databases

Both the genomic sequences and annotation information used in this study were downloaded from the Pathosystems Resouce Integration Center (PATRIC, http://www.patricbrc.org) [36] on March 8, 2012. The detailed information of these genomes is listed in Table S1.

### Reconstruction of the Streptococcal Species Tree

As did in previous study [7], we select *Lactococcus lactis* as an out-group species to reconstruct the *Streptococcus* phylogeny. To build a super multi-gene matrix for a better resolution of phylogeny, we used the Proteinortho v4.26 [52] to identify a maximal number of orthologous genes that are shared by 38 streptococcal genomes and *Lactococcus lactis*. A total of 530 single-copy protein-coding genes were finally obtained. For each gene, its amino acid sequences extracted from the 39 genomes were concatenated and aligned by using the ClustalW program. The maximum likelihood (ML) analysis was then performed using PhyML v3.0.1 [53] to reconstruct the species tree. The reliability of internal nodes of the tree was assessed by aLRT SH-like branch support.

### Searching for Sequences that Encode Histidine Triad Motifs

To identify genes containing HTP domains in *Streptococcus*, both BLAST and HMM (hidden Markov model) searching methods were adopted. The amino acid sequence of the HTP domain in *Pht*D was first used as query sequence to Blast against the protein database of all streptococcal genomes. The amino acid sequences of hits obtained were then used one by one to Blast against all genomes for any undetected homologs. The threshold for the E-value was set to 1.0 to find a maximal number of candidate genes. In the HMM procedure, the HTP domain model (PF04270.8) was downloaded from the Pfam database and served as the search query. The hits obtained from the two methods were consolidated together to remove redundant hits. All remaining non-redundant genes were tested by Pfam analysis to remove sequences that did not contain the HTP domain.

### Alignment and Phylogenetic Analysis of *htp* Genes

Multiple alignments of amino acid sequences were performed using the ClustalW program integrated in mega 5.0 [54], [55] with default parameters. The resulting amino acid sequence alignments were then used to guide the alignments of nucleotide coding sequences. Phylogenetic trees were constructed based on either the alignments of CDS or amino acid sequences by using PhyML v3.0.1 [53]. The reliability of internal nodes was assessed by aLRT SH-like branch support.

### Calculation of Nucleotide Substitution Rate and Recombination Tests

To evaluate the relative rate of evolution of the two *htp* subfamilies, the synonymous substitution rates (Ks) for the full-length *htp* genes, their neighboring *lmb* genes and housekeeping genes were calculated using mega 5.0 with the Nei and Gojobori model [55]. Eight housekeeping genes were selected in this study, including *dna*E (encoding DNA polymerase III), *glt*X (encoding glutamyl-tRNA synthetase), *pyr*E (encoding orotate phosphoribosyltransferase), *rec*A (encoding recombinase A protein), *rpo*B (encoding the DNA-directed RNA polymerase beta chain), *sec*A (encoding preprotein translocase subunit SecA), *sec*Y (encoding preprotein translocase subunit SecY) and *sod*A (encoding superoxide dismutase). All of these genes have been separately or collectively used in phylogenetic analysis of *Streptococcus* [7], [10], [56], [57]. To detect the evolutionary constraints on the functional domains, the Ks of HTP and LRR domains were also calculated. A sliding-window analysis of the ratio of non-synonymous to synonymous substitution rates (Ka/Ks) was performed on the sequences of the two subfamilies using DnaSP (http://www.ub.edu/dnasp) [39], with a window size of 60 bases and a step size of 15 bases. Recombinant events among *htp* genes were detected with the RDP v3.34 software [58] using four automated recombination detection methods, including RDP, Genconv, Chimaera and Maximum Chi Square, with default parameters.

## Supporting Information

Figure S1 **Physical map of all identified** ***htp*** **genes in 38 streptococcal genomes.**
(TIF)

Figure S2 **The phylogenetic tree of** ***htp*** **genes based on amino acid sequences.**
(TIF)

Figure S3 **Schematic representation of the recombination events occurring among** ***htp*** **genes identified from streptococcal species.** Each line represents a recombinant sequence, and the red boxes below indicate the sequences exchanged from homologs. The name of the putative parental species for each fragment is indicated.
(TIF)

Table S1 **Detailed information and** ***htp*** **copy numbers for all genomes used in this study.**
(XLS)
