# Supplementary figures and images for: Insight into the Evolution of the Histidine Triad Protein (HTP) Family in Streptococcus
Source: PLoS One. 2013 Mar 20;8(3):e60116. doi: 10.1371/journal.pone.0060116 (PMC3603884; doi:10.1371/journal.pone.0060116)

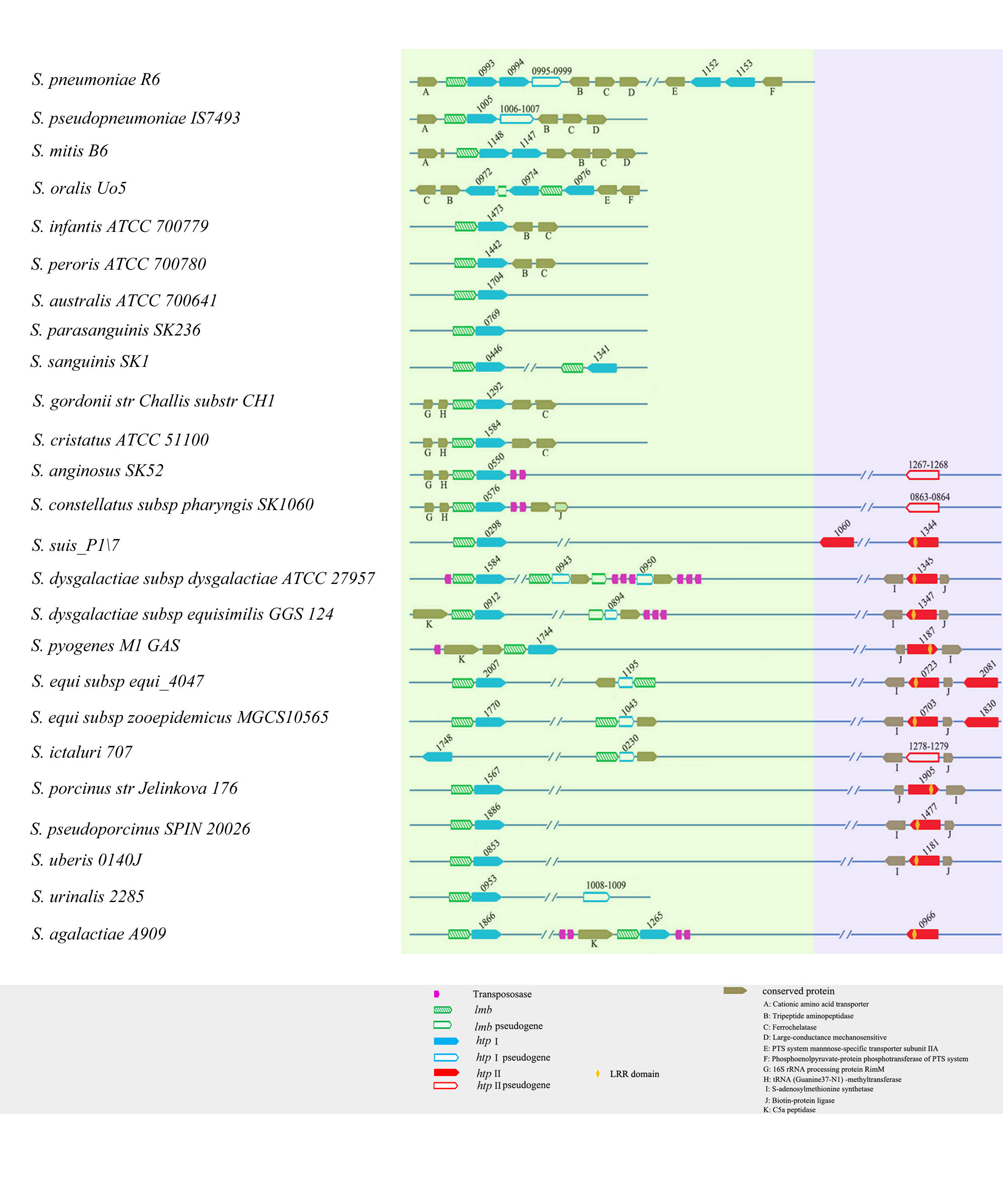

Supplement: Figure S1 — Physical map of all identified htp genes in 38 streptococcal genomes. (TIF) [file pone.0060116.s001.tif]

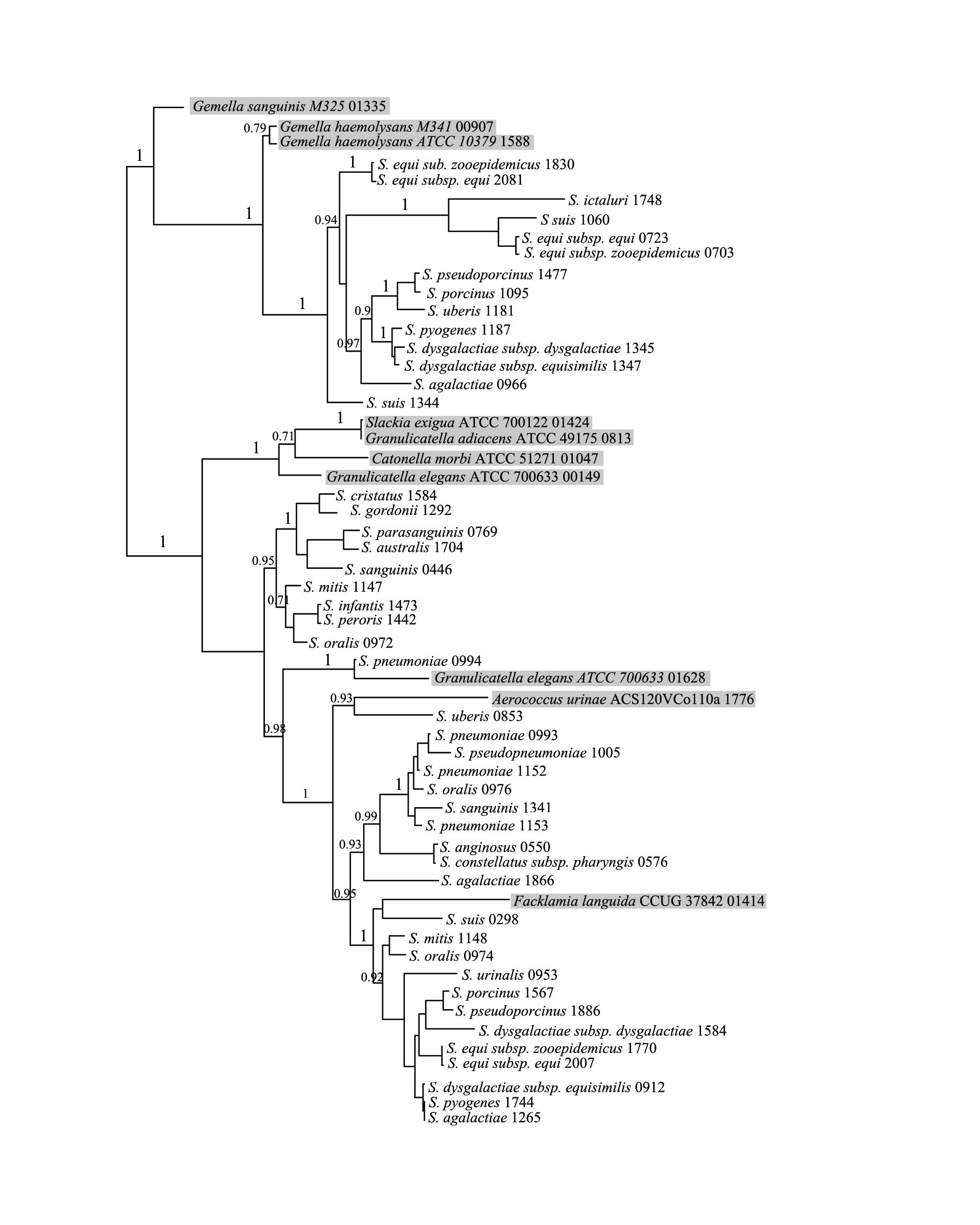

Supplement: Figure S2 — The phylogenetic tree of htp genes based on amino acid sequences. (TIF) [file pone.0060116.s002.tif]

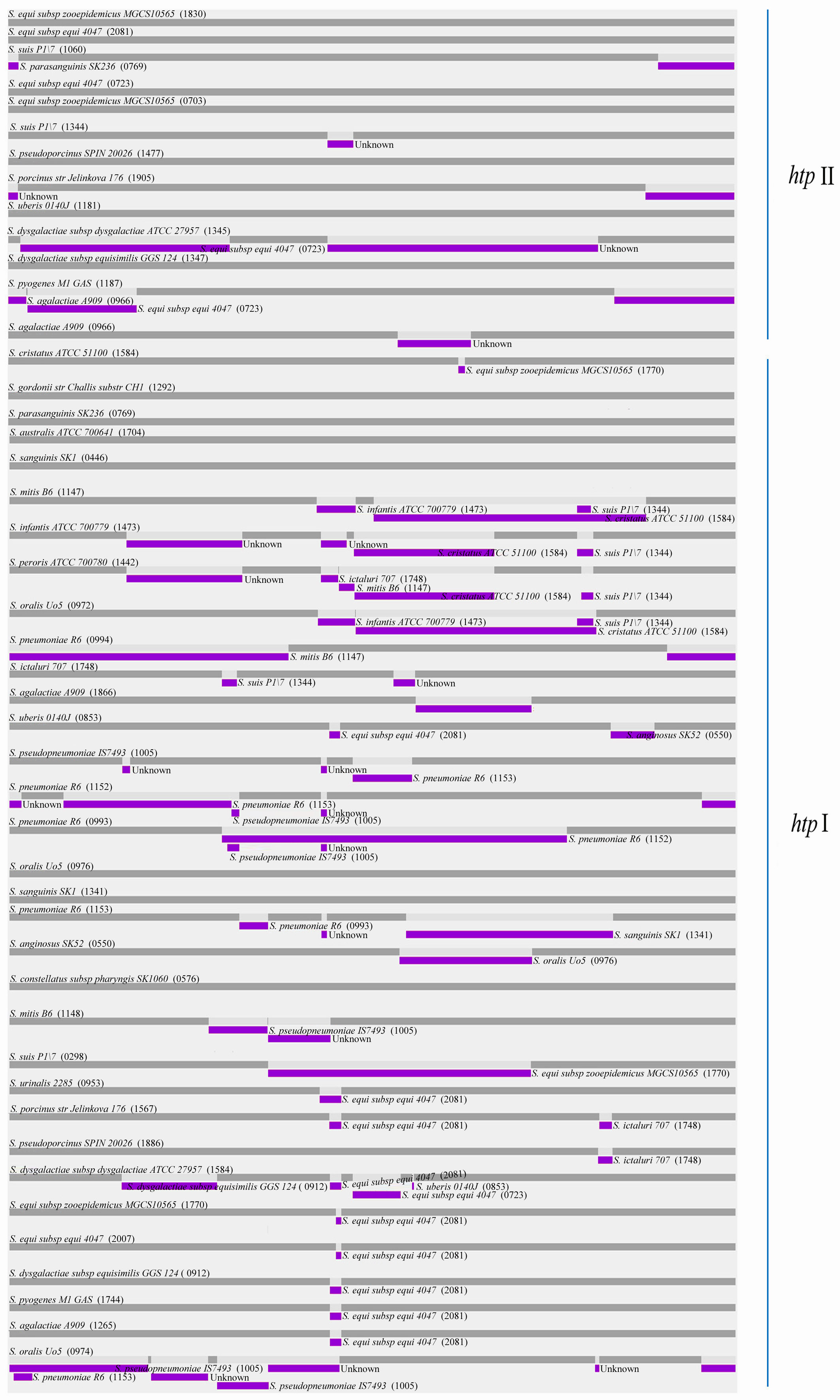

Supplement: Figure S3 — Schematic representation of the recombination events occurring among htp genes identified from streptococcal species. Each line represents a recombinant sequence, and the red boxes below indicate the sequences exchanged from homologs. The name of the putative parental species for each fragment is indicated. (TIF) [file pone.0060116.s003.tif]
